# Green synthesis, characterization, and anticancer activity of silver nanoparticles via *Pulsatilla koreana* root extract

**DOI:** 10.3389/fnut.2026.1746280

**Published:** 2026-02-16

**Authors:** Yaxi Han, Ling Zhu, Xindi Zhang, Kunlun Wang, Dixin Sha, Qing Chen, Xinmiao Yao, Ye Zhou, Bo Li, Lijun Guan

**Affiliations:** 1 Institute of Food Processing, Heilongjiang Academy of Agricultural Sciences, Harbin, China; 2 Key Laboratory of Food Processing of Heilongjiang Province, Harbin, China

**Keywords:** anticancer activity, antimicrobial activity, functional food, green synthesis, *Pulsatilla koreana*, silver nanoparticles

## Abstract

A green synthesis route for silver nanoparticles (Pk-AgNps) has been successfully developed using *Pulsatilla koreana* root extract as both reducing and stabilizing agent. Optimal synthesis conditions of 80 °C for 60 min produced nanoparticles characterized by a distinct surface plasmon resonance (SPR) peak at 464 nm. Comprehensive characterization through field-emission transmission electron microscopy (FE-TEM), X-ray diffraction analysis (XRD), and Fourier transform infrared analysis (FTIR) confirmed the formation of quasi-cubic, polycrystalline nanoparticles in the size range of 80–100 nm, stabilized by phytochemical constituents from the extract. The synthesized Pk-AgNps demonstrated significant bioactivities including concentration-dependent antioxidant capacity and potent antibacterial effects against both Gram-positive and Gram-negative bacteria. Furthermore, the nanoparticles exhibited selective cytotoxicity against human cancer cell lines, with particular efficacy against A549 lung cancer cells. Mechanistic studies revealed that this anticancer activity involves Reactive oxygen species (ROS)-mediated apoptosis through suppression of the EGFR/MAPK signaling pathway and regulation of Bcl-2/Bax expression. These multifunctional properties of Pk-AgNps as a promising candidate for further exploration in functional food and biomedical applications.

## 1 Introduction

The growing consumer demand for natural and functional foods has intensified research into bioactive ingredients derived from edible and medicinal plants (1). In the food industry, there is a particular interest in developing natural antimicrobial (2) and antioxidant (3) agents to enhance food preservation and safety, as well as to create novel functional foods (4). Nanotechnology offers promising tools for this endeavor, with silver nanoparticles (AgNps) being one of the most studied nanomaterials due to their potent bioactivities (5).

Conventional methods for synthesizing AgNps typically rely on physical and chemical processes, each presenting significant drawbacks. Physical approaches, such as laser ablation and ball milling, avoid the use of chemical solvents but often come with high energy consumption, low yield, and poor control over particle size and morphology (6). The more commonly employed chemical reduction methods frequently rely on toxic reducing agents like sodium borohydride and stabilizing chemicals, which can introduce cytotoxic residues. Furthermore, these methods require complex post-synthesis purification and surface modification steps to remove reaction by-products and unreacted precursors—a process that is often challenging and incomplete (7). The potential presence of these harmful residuals poses a major barrier to the adoption of such nanoparticles in food-related applications, raising substantial safety concerns and environmental issues (8, 9). Consequently, the development of green, eco-friendly synthesis methodologies has become a paramount objective (10). The use of plant extracts for the biogenic synthesis of AgNps has emerged as a superior alternative (11), where phytoconstituents such as polyphenols (12), flavonoids (13), and terpenoids (14) naturally present in the plant matrix serve as effective reducing and capping agents (15). Numerous plant species have been successfully employed for the green synthesis of AgNps with demonstrated antimicrobial efficacy. For instance, green tea (*Camellia sinensis*) extract, rich in epigallocatechin gallate, has been utilized to synthesize AgNps ranging from 4 to 33 nm, exhibiting potent antibacterial activity against foodborne pathogens (16). Similarly, *Origanum vulgare* (oregano) and *Lavandula angustifolia* (lavender) extracts have demonstrated superior antibacterial performance against both Gram-positive and Gram-negative bacteria, attributed to their high antioxidant activity and total phenolic content (17). Furthermore, fruit extracts from *Viburnum opulus* (Gilaburu), containing abundant polyphenols and organic acids, have been successfully employed to synthesize AgNps with excellent antimicrobial properties suitable for food safety applications (18). *Ziziphus spina-christi* leaf extract has also been reported to produce spherical AgNps (average size 24 nm) within 30 min, demonstrating reliable antibacterial and antifungal activity against common human pathogens (19). This approach not only aligns with the principles of green chemistry but also offers a cost-effective and scalable pathway for producing nanoparticles that are more suitable for consideration in food contact applications.

The selection of an appropriate plant source is critical to the success of this green synthesis strategy. Ideal candidates are those with a history of use, recognized bioactivity, and a rich profile of reducing compounds (20, 21). *Pulsatilla koreana*, a plant with a history of use in traditional medicine for its anti-inflammatory and anti-cancer properties (22). These therapeutic effects are largely attributed to its bioactive compounds, particularly triterpenoid saponins, in its roots (23). These compounds are known for their surface-active and reduction properties (24, 25), suggesting their potential as effective agents for the biogenic synthesis of metal nanoparticles. From a functional food perspective, plant extracts like that from *P. koreana* can be considered as a source of ‘green’ functional ingredients for nanomaterial fabrication. However, its application in the synthesis of AgNps, and particularly the characterization of the resulting particles for potential use in food-related applications, remains underexplored.

This study aims to develop a green and sustainable synthesis route for silver nanoparticles using an aqueous extract from *Pulsatilla koreana* roots (Pk-AgNps). The biosynthesis of AgNps using plant extracts has been extensively explored as a sustainable alternative to conventional methods, with numerous studies reporting their antimicrobial and antioxidant activities. *P. koreana* is well-documented for its rich profile of bioactive compounds, including ginsenosides and flavonoids, suggesting its potential to impart distinct functionalities to the synthesized nanoparticles. Accordingly, the synthesized nanoparticles were systematically characterized by a suite of analytical techniques, including UV–Visible (UV–Vis) spectroscopy, FE-TEM, energy-dispersive X-ray spectroscopy (EDX), selected area electron diffraction pattern (SAED), XRD, dynamic light scattering particle size analysis (DLS) and FTIR. The functional profiling of Pk-AgNps extended to multiple bioactivity assessments, encompassing the evaluation of antioxidant potential via 2,2-diphenyl-1-picrylhydrazyl (DPPH) scavenging, antimicrobial activity against *Escherichia coli* and *Staphylococcus aureus*, and *in vitro* anticancer activity. While the direct therapeutic application falls outside strict food science, assessing such potent bioactivities provides valuable initial insights into the broader biological potential and mechanism of action of these nanoparticles, which could inform future research into their functional effects and safety profile. This work seeks to contribute to the exploration of plant-derived extracts as versatile tools for producing functional nanomaterials, with implications for future applications in the food and health industries.

## 2 Materials and methods

### 2.1 Materials

Dried *P. koreana* roots were obtained from Hanbang Bio Laboratory, Kyung Hee University (Republic of Korea). Silver nitrate (AgNO_3_, Cat. No. 31630), DPPH, gallic acid, 3-(4, 5-dimethyl-2-thiazolyl)-2, 5-diphenyl-2H tetrazolium bromide (MTT, Cat. No. 475989), 2′,7′-dichlorodihydro-fluorescein diacetate (DCFH-DA, Cat. No. D6883), TRIzol reagent® (Cat. No. T9424), and Hoechst-33258 (Cat. No. 94403) were sourced from Sigma-Aldrich Chemicals (St. Louis, MO, USA). Human lung carcinoma (A549), human breast cancer (MCF7), and human gastric adenocarcinoma (AGS) cell lines came from the Korean Cell Line Bank (KCLB, Seoul, Republic of Korea). Cell culture media and supplements, including RPMI 1640, fetal bovine serum (FBS), phosphate-buffered saline (PBS), and penicillin–streptomycin, were procured from GenDEPOT (Barker, TX, USA). All chemicals were analytical grade and used without further purification.

### 2.2 Green synthesis of Pk-AgNps

Briefly, Pk-AgNps were synthesized using an aqueous extract of *P. koreana* roots. The extract was prepared by autoclaving 10 g finely ground root powder suspension at 100 °C for 1 h, followed by filtration and storage at 4 °C for further use (26). For nanoparticle green synthesis, the extract was mixed with AgNO_3_ solution was added dropwise to the *P. koreana* extract (10%, *v*/*v*) to achieve a final concentration of 1 mmol/L AgNO_3_ in the reaction mixture. The mixture was then incubated at 80 °C in an oil bath to facilitate the reduction of Ag^+^ to Ag^0^ atoms. The formation of silver nanoparticles was indicated by a distinct color change in the reaction solution, which was subsequently confirmed by UV–Vis spectroscopy (2,100 Pro; Amersham Biosciences). Following synthesis, the Pk-AgNps were purified through repeated centrifugation at 16,000 rpm for 15 min followed by washing with distilled water. The resulting pellet was air-dried overnight to obtain powdered form for subsequent characterization (27).

### 2.3 Characterization of Pk-AgNps

The formation and optical properties of the synthesized nanoparticles were verified by UV–Vis spectroscopy (300–800 nm) (28). Spectra were recorded using 200 μL aliquots in quartz cuvettes with 1 cm path length. Morphological and structural characterization was performed using a JEM-2100F field emission transmission electron microscope (JEOL, Peabody, MA) operating at 200 kV. Aqueous suspensions of nanoparticles were deposited onto carbon-coated copper grids and dried at 60 °C for 20 min prior to analysis. FE-TEM imaging revealed nanoparticle morphology and size distribution. Elemental composition and purity were assessed by EDX, while elemental mapping confirmed the spatial distribution of constituent elements (29). Crystalline structure was determined by SAED (30).

XRD analysis was obtained with a D8 Discover diffractometer (Bruker, Hamburg, Germany) equipped with a Cu-Kα radiation source (*λ* = 1.54 Å). Prior to analysis, the powdered samples were prepared by grinding in an agate mortar to ensure homogeneity, then uniformly packed into a glass sample holder. Measurements were conducted over a 2*θ* range of 20–80° at 6°/min with an interval of 0.02°, operating at 40 kV and 40 mA. The average crystallite size was calculated using the Debye–Scherrer equation, which correlates the broadening of the XRD peaks with the size of the crystalline domains:

$$D=\frac{0.9\lambda }{\beta \cos\theta }$$ (1)

where *D* is the crystallite size in nm, *λ* is the wavelength of Cu-Kα radiation in nm, *β* is the full width at half maximum (FWHM) in radians, and *θ* is half of the Bragg angle in radians (31).

The hydrodynamic diameter, polydispersity index (PDI), and zeta potential of the nanoparticles were determined by DLS using an ELSZ-2000 particle size analyzer (Otsuka Electronics, Japan). Prior to measurement, the nanoparticle suspension was appropriately diluted with deionized water to achieve an optimal scattering intensity. The diluted sample was then transferred into a clean, disposable polystyrene cuvette for size and PDI measurements, which were conducted at a fixed scattering angle of 90° and a stabilized temperature of 25 °C zeta potential was measured using a dedicated folded capillary cell under the same temperature conditions. Data were collected and analyzed to obtain size distribution profiles based on intensity, number, and volume (32), providing crucial information about the colloidal stability and size homogeneity of the nanoparticle suspensions.

Surface functional groups of the biogenic Pk-AgNps were characterized by FTIR spectroscopy using a Perkin Elmer Spectrum One spectrometer (PerkinElmer Inc., Waltham, MA, USA). Spectra were collected over the range of 4,000–450 cm^−1^ at a resolution of 4 cm^−1^ to identify the biomolecules involved in nanoparticle stabilization (33).

### 2.4 DPPH radical scavenging assay

The antioxidant activity of the biosynthesized Pk-AgNps was evaluated using the DPPH radical scavenging assay (26, 34). In brief, 20 μL of each sample solution was mixed with 180 μL of 1 mM DPPH methanolic solution. Different concentrations of Pk-AgNps and gallic acid (as positive control) were prepared for comparison. The reaction mixtures were protected from light and incubated at room temperature for 30 min. Absorbance was then measured at 517 nm using a UV–Vis spectrophotometer. The DPPH radical scavenging activity was calculated according to equation:

$$\text{DPPH radical scavenging}=\frac{A_{\text{Control}}-A_{\text{Sample}}}{A_{\text{Control}}}\times 100\%$$ (2)

where *A*_Control_ represents the absorbance of the control (distilled water), and *A*_Sample_ denotes the absorbance of tested samples.

#### 2.4.1 Antimicrobial activity of Pk-AgNps

The antimicrobial efficacy of Pk-AgNps were assessed against pathogenic microorganisms *E. coli* and *S. aureus* using the disk diffusion assay. Bacterial suspensions were prepared by diluting overnight cultures in lysogeny broth to an optical density (OD) of 0.1. Aliquots of 100 μL from each bacterial suspension were uniformly spread on Mueller–Hinton agar (MHA) plates. Sterile paper disks were impregnated with 30 μL of freshly prepared PK-AgNp suspensions at concentrations of 15, 30, and 45 μg/disk, while Neomycin (30 μg/disk, N30) served as the positive control. All plates were incubated at 37 °C for 24 h. Following incubation, the inhibition zones surrounding each disk were measured and recorded (35).

Based on the disk diffusion results, the minimum inhibitory concentration (MIC) of Pk-AgNps against *E. coli* and *S. aureus* was further determined using the broth dilution method. MIC is defined as the lowest concentration of an agent that inhibits visible microbial growth [18, 19]. Briefly, a series of liquid cultures containing Pk-AgNps at concentrations ranging from 1–100 μg/mL were prepared in LB medium. Each tube was inoculated with 10 μL of a freshly prepared bacterial suspension (≈10^8^ CFU/mL) of either *E. coli* or *S. aureus*. The cultures incubated at 37 °C for 24 h with shaking at 250 rpm. After incubation, bacterial growth was assessed by measuring the OD_600_ using a UV–Vis spectrophotometer. The negative controls containing LB media without nanoparticles was included to represent uninhibited microbial growth. All experiments were carried out in triplicate, with results expressed as mean ± SD.

### 2.5 Cell culture

A549, MCF7, and AGS human cancer cell lines were independently cultured in RPMI-1640 medium supplemented with 10% FBS to provide essential nutrients and growth factor, and 1% Penicillin–Streptomycin to prevent microbial contamination. The cells were maintained at 37 °C in a humidified atmosphere containing 5% CO_2_, with medium refreshed every 2–3 days to ensure optimal growth conditions.

### 2.6 Cell viability assay

*In vitro* cytotoxicity of Pk-AgNps was evaluated using MTT assay (36). Cells were seeded in a 96-well plate (Corning Costar, Lowell, NY, USA) at a density of 1 × 10^4^ cells/well and allowed to adhere overnight. Subsequently, the cells were treated with varying concentrations of Pk-AgNps (0, 1, 5, 10, 25, 50, and 100 μg/mL) for 24 h at 37 °C. After treatment, MTT assay was performed by adding 10 μL of MTT stock solution (5 mg/mL in PBS) to each well, yielding a final concentration of 0.5 mg/mL in the culture medium, followed by incubation at 37 °C for 3 h. The resulting formazan crystals were dissolved by 200 μL of DMSO and absorbance was measured at 570 nm using a microplate reader (Bio-Tek Instruments, Inc., Winooski, VT, USA). Cell viability was calculated relative to untreated control cells, which were considered to represent 100% viability. All experiments were performed in triplicate, with data presented as mean ± SD.

### 2.7 ROS generation

Intracellular ROS levels were measured using the fluorescent probe DCFH-DA. A549, MCF7, and AGS cells were seeded in 96-well black plates with clear bottoms (Corning Costar, Lowell, NY, USA) and allow to adhere. After 24 h exposure to variety concentrations of Pk-AgNps (0, 1, 5, 10 μg/mL), the cells were loaded with 100 μL of DCFH-DA solution, yielding a final concentration of 15 μmol/L, and incubated for 30 min in the dark condition. Fluorescence intensity, corresponding to intracellular ROS levels, was quantified using a Synergy™2 microplate reader with excitation and emission wavelengths set at 485 nm and 535 nm, respectively. The increase in fluorescence signal reflects the oxidation of DCFH-DA to fluorescent DCF by intracellular ROS (37).

### 2.8 Apoptosis detection

Apoptosis nuclear morphological changes induced by Pk-AgNps were evaluated using Hoechst-33258 staining (38). A549, MCF7, and AGS cells were cultured in 6-well plates (SPL Life Sciences Co., Ltd., Korea) at a density of 1 × 10^5^ cells/well and cultured overnight. Following 24 h treatment with 10 μg/mL Pk-AgNps, cells were washed gently with 1 × PBS and fixed with 3.7% formaldehyde for 10 min at room temperature. After too additional 1 × PBS washes, cells were stained with 5 μg/mL Hoechst-33258 solution for 30 min under dark conditions. Nuclear morphology was examined using fluorescence microscope (Carl Zeiss, Axiovert 200 M, Oberkochen, Germany), with apoptotic cells identified by characteristic chromatin condensation and nuclear fragmentation. Scale bars were added by using ImageJ software.

### 2.9 Real-time quantitative reverse transcription-PCR (qRT-PCR) analysis

Gene expression analysis was performed on A549, MCF7, and AGS cell lines treated and non-treated of 10 μg/mL Pk-AgNps for 24 h. The total RNA was extracted using TRIzol reagent® according to the manufacturer’s protocol. cDNA synthesis was carried out with 500 ng of total RNA using oligo (dT) 15 primer (0.2 mM), and AMV Reverse Transcriptase (10 units/μL) from Superscript™ First-Strand Synthesis Kit (Cat. No. 18091050, Invitrogen, Carlsbad, CA). qRT-PCR was performed in 96-well plate using 100 ng of cDNA in a 20 μL reaction volume containing SYBR® Green SensiMix Plus Master Mix (Quantace, Taunton, MA). The thermal cycling conditions were as follows: initial denaturation at 95 °C for 10 min, followed by 40 cycles of 95 °C for 10 s, 58 °C for 10 s, and 72 °C for 20 s. Melting curve analysis confirmed amplification specificity by demonstrating single product formation. Fluorescent data were collected at the extension step of each cycle, and threshold cycle (Ct) values were determined using the 2^−ΔΔCt^ method (39). *GAPDH* served as the reference gene for normalization, and all primer sequences are listed in Table 1. Experiments were conducted in triplicate, with statistical significance assessed using Student’s *t*-test.

**Table 1:** Primer sequences used for gene expression analysis by qRT-PCR.

Primer	Sequence
*Bcl-2*	Forward: 5′-AAT GGG CAG CCG TTA GGA AA-3’
	Reverse: 5′-GCG CCC AAT ACG ACC AAA TC -3’
*Bax*	Forward: 5′-GGA TGC GCT GAA ACG TGG A-3’
	Reverse: 5′-CAG GAA TGA GTA CAC GAA GCC-3’
*EGFR*	Forward: 5′-CCA ACC AAG CTC TCT TGA GG-3’
	Reverse: 5′-GCT TTC GGA GAT GTT GCT TC-3’
*ELK-1*	Forward: 5′-TGA GCT GTA GGG AAA CGC AG-3’
	Reverse: 5′-CAG GGG TAC CTG TGT GTA GC-3’
*MAPK14*	Forward: 5′-CGA CTT GCT GGA GAA GAT GC-3’
	Reverse: 5′-TCC ATC TCT TCT TGG TCA AGG-3’
*GAPDH*	Forward: 5′-CAA GGT CAT CCA TGA CAA CTT TG-3’
	Reverse: 5′-GTC CAC CAC CCT GTT GCT GTA G-3’

### 2.10 Statistical analysis

Results are expressed as mean ± SD. Statistical analyses were performed using GraphPad Prism 9 software (La Jolla, CA, USA). Differences among multiple groups were evaluated by one-way ANOVA followed by a post-hoc test for pairwise comparisons when overall significance was detected. All experiments were performed at least in triplicate (*n* ≥ 3) unless stated otherwise. Statistical significances between control and sample groups were evaluated by Student’s *t*-test with two-tailed distribution and two-sample equal variances. A greater extent of statistical significances was assigned with increasing number of asterisks (^*^p < 0.05, ^**^p < 0.01, ^***^p < 0.001).

## 3 Results and discussions

### 3.1 Synthesis of Pk-AgNps

To optimize the synthesis protocol, the effects of reaction temperature and time on nanoparticle formation were systematically investigated. The reaction progress was monitored both visually and by UV–Vis spectroscopy.

Visually, the formation of Pk-AgNps was indicated by a distinct color change of the reaction mixture from pale yellow to deep brown. This color transformation, which results from the excitation of SPR in the metal nanoparticles (28), was most pronounced in the sample reacted at 80 °C for 60 min. The intensity of the brown color was noticeably weaker at lower temperatures (20 °C and 40 °C) and shorter reaction times at 80 °C (15 and 30 min), suggesting a slower and less complete reduction process under these conditions.

**Figure 1:**
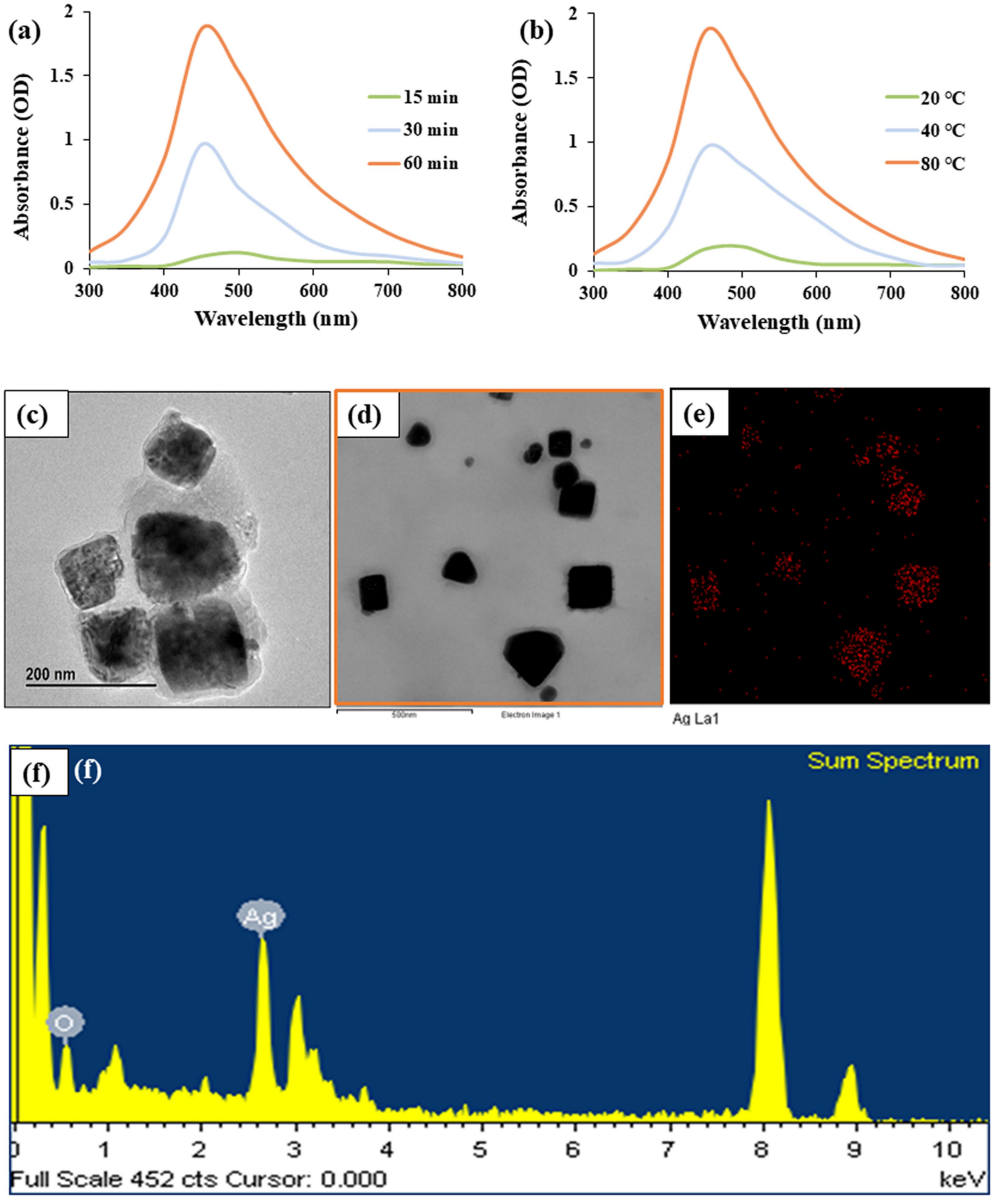
UV–Vis spectra monitoring the formation of Pk-AgNps under reaction time **(a)** and temperature **(b)**. FE-TEM image revealing nanoparticle morphology **(c, d)**. Elemental mapping **(e)** and EDX spectrum **(f)** presenting the elemental distribution and composition, respectively.

The formation and optimization of Pk-AgNps were unequivocally confirmed by UV–Vis spectroscopy. The aqueous suspensions of the reaction products obtained under different conditions exhibited characteristic absorption peaks in the range of 450–470 nm, which is typical for the SPR of spherical silver nanoparticles (40). As illustrated in Figure 1a, the intensity of the SPR peak was strongly dependent on both reaction temperature and time. At a fixed reaction time of 1 h, increasing the temperature from 20 °C to 80 °C led to a significant enhancement in peak intensity and a sharper profile, with the maximum absorbance observed at 80 °C. Similarly, for the reaction conducted at 80 °C, the peak intensity increased progressively with time from 15 to 60 min, reaching a maximum at 60 min. The most intense and well-defined SPR peak, centered at approximately 464 nm, was obtained under the condition of 80 °C for 60 min (Figures 1a,b). This combination of temperature and time was therefore identified as optimal for the efficient synthesis of Pk-AgNps as the optimum factors for all subsequent experiments.

The observed dependence on temperature and time can be attributed to the kinetics of the reduction reaction. Higher thermal energy likely facilitates the release of active reducing phytoconstituents, such as polyphenols and triterpenoid saponins from the root extract, and accelerates the electron transfer process necessary for the reduction of Ag^+^ to Ag^0^ (41, 42). The increased peak intensity and stability at 80 °C over 60 min indicate a higher yield and better formation of nanoparticles. The broad nature of the SPR peak, even under optimal conditions, suggests a distribution of particle sizes, which is common in green synthesis approaches due to the complex mixture of capping agents present in the plant extract (43).

### 3.2 Characterization of Pk-AgNps

#### 3.2.1 FE-TEM analysis

The morphology and size of the synthesized Pk-AgNps were elucidated using FE-TEM (44). As shown in Figures 1c,d, the nanoparticles are predominantly irregular quasi-cubic in shape, with a size distribution ranging from approximately 80–100 nm. This non-spherical morphology is consistent with the broad absorption peak observed in the UV–Vis spectrum, as anisotropic particles often exhibit broader SPR bands. Elemental mapping was performed to assess the distribution of elements within the nanoparticles. The maps for silver (Ag) presented in Figures 1d,e demonstrate a homogeneous distribution of the element across the entire scanned area of the nanoparticles, confirming that the observed structures are indeed composed of silver.

Furthermore, the compositional purity of the Pk-AgNps was verified by EDX. The EDX spectrum (Figure 1f) shows a strong and characteristic optical absorption peak for metallic silver at approximately 2.7 keV (the copper grid recorded at approximately 8 keV), indicates that the synthesized Pk-AgNps are of high purity. The minor carbon and oxygen signals, which are not explicitly discussed here, can typically be attributed to the thin layer of biomolecular capping agents derived from the *P. koreana* root extract that is stabilizing the nanoparticles (45).

#### 3.2.2 Crystallographic analysis by SAED and XRD

**Figure 2:**
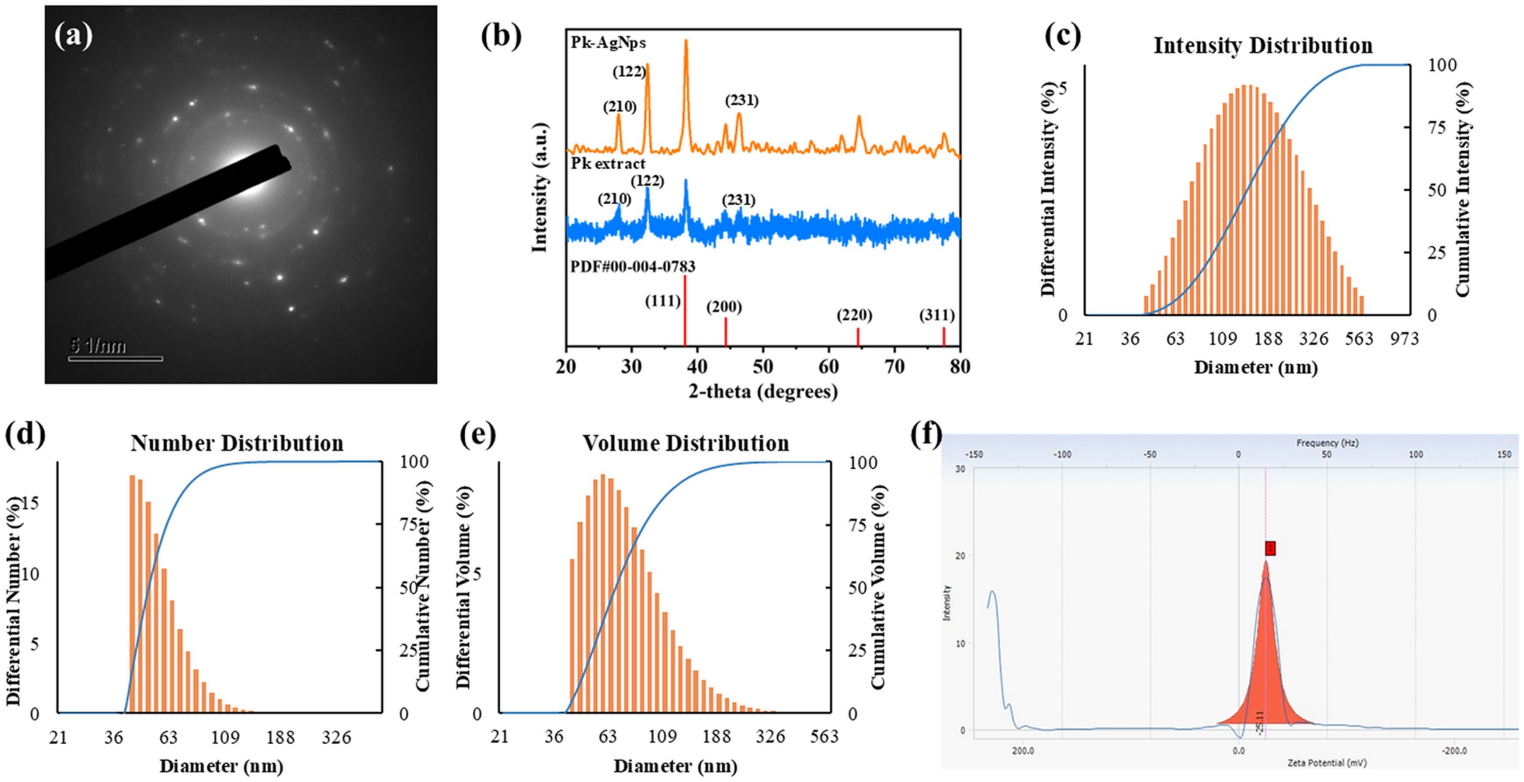
SAED pattern **(a)** and XRD spectrum **(b)** collectively verify the crystalline structure of the nanoparticles. The hydrodynamic diameter and size polydispersity were assessed by DLS, showing distribution profiles based on intensity **(c)**, number **(d)**, and volume **(e)**. The surface charge characteristic, indicated by the zeta potential measurement **(f)**.

The crystalline nature and phase composition of the biosynthesized Pk-AgNps were determined using XRD. The XRD pattern, as shown in Figure 2b, exhibited distinct diffraction peaks at 2*θ* values which were indexed to the (210), (122), (111), (200), (231), (220), and (311) lattice planes. The presence of these well-defined peaks confirms that the synthesized nanoparticles are crystalline. The diffraction pattern predominantly aligns with the face-centered cubic (FCC) crystal structure of metallic silver.

The diffraction pattern matches predominantly the face-centered cubic (FCC) crystal structure of metallic silver (PDF card no. #00-004-0783), with the (111), (200), (220), and (311) planes being characteristic of this structure (46). However, the appearance of additional peaks, including (210), (122), and (231), which are atypical for pure FCC silver, suggests the formation of crystalline phases associated with the capping agents (47, 48). These extra reflections are likely attributable to organic biomolecules from the *P. koreana* root extract that are firmly anchored to the surface of the silver nanoparticles. Notably, the positions of the (210), (122), and (231) peaks align with diffraction features of the extract itself. During the green synthesis process, these phytochemicals (e.g., saponins, flavonoids) not only function as reducing agents for silver ions but can also form an ordered crystalline layer on the nanoparticle surface. The coexistence of diffraction signals from both the metallic silver core and the bio-organic shell is a common phenomenon in plant-mediated nanoparticles and corroborates the role of the extract as a stabilizing agent, as inferred from the FTIR analysis.

The crystalline structure was further corroborated by SAED in the TEM. The SAED pattern (Figure 2a) displayed concentric rings composed of discrete spots, which can be indexed to the (111), (200), (220), and (311) planes of FCC silver. This pattern confirms the polycrystalline nature of the Pk-AgNps.

#### 3.2.3 Size distribution measurements and zeta potential

The hydrodynamic diameter and size distribution of the Pk-AgNps in suspension were further assessed by DLS. The analysis presented three distinct distribution profiles based on intensity, number, and volume (Figures 2c–e). The Z-average hydrodynamic diameter was 244.4 ± 6.5 nm, with a PDI value of 0.237. According to recent literature, a PDI value below 0.3 is considered indicative of a narrow size distribution and homogeneous nature for metallic nanoparticles, which is acceptable for various applications (49, 50). The zeta potential of Pk-AgNps was measured at −25.11 mV ± 1.46 mV (Figure 2f).

A notable discrepancy was observed between the particle sizes obtained from FE-TEM (80–100 nm) and DLS (Z-average of 244.4 ± 6.5 nm). This difference is commonly reported and can be attributed to the fundamental distinctions in the measurement principles of these two techniques (51). FE-TEM provides direct visualization of the primary metallic core under high vacuum and in a dehydrated state, revealing the actual physical dimensions of the nanoparticles. In contrast, DLS measures the hydrodynamic diameter of particles in their native aqueous suspension (52). This hydrodynamic size encompasses not only the metallic core but also the layer of biomolecules (such as saponins and polyphenols) from the *P. koreana* root extract adsorbed onto the nanoparticle surface, as well as the associated solvation shell that moves with the particle during Brownian motion. Furthermore, DLS is inherently more sensitive to larger particles in a population due to the intensity-weighted nature of the measurement, and can be influenced by even minor aggregation events, which would significantly increase the apparent hydrodynamic diameter. The obtained PDI of 0.237 (<0.3) indicates a relatively narrow and uniform size distribution, which is commonly achieved for plant-mediated synthesized nanoparticles; moreover, the unimodal intensity distribution profile (Figure 2c) confirms the absence of large aggregates or multiple particle populations (53). The zeta potential value of −25.11 ± 1.46 mV further confirms a moderate negative surface charge, which contributes to colloidal stability through electrostatic repulsion (54). Therefore, the larger hydrodynamic size and broader distribution reported by DLS are consistent with the presence of a stabilizing biological corona derived from plant phytochemicals, and confirm that the Pk-AgNps remain well-dispersed in the aqueous phase, which is a critical factor for their potential application in liquid-based systems (55).

#### 3.2.4 FTIR spectroscopic analysis

**Figure 3:**
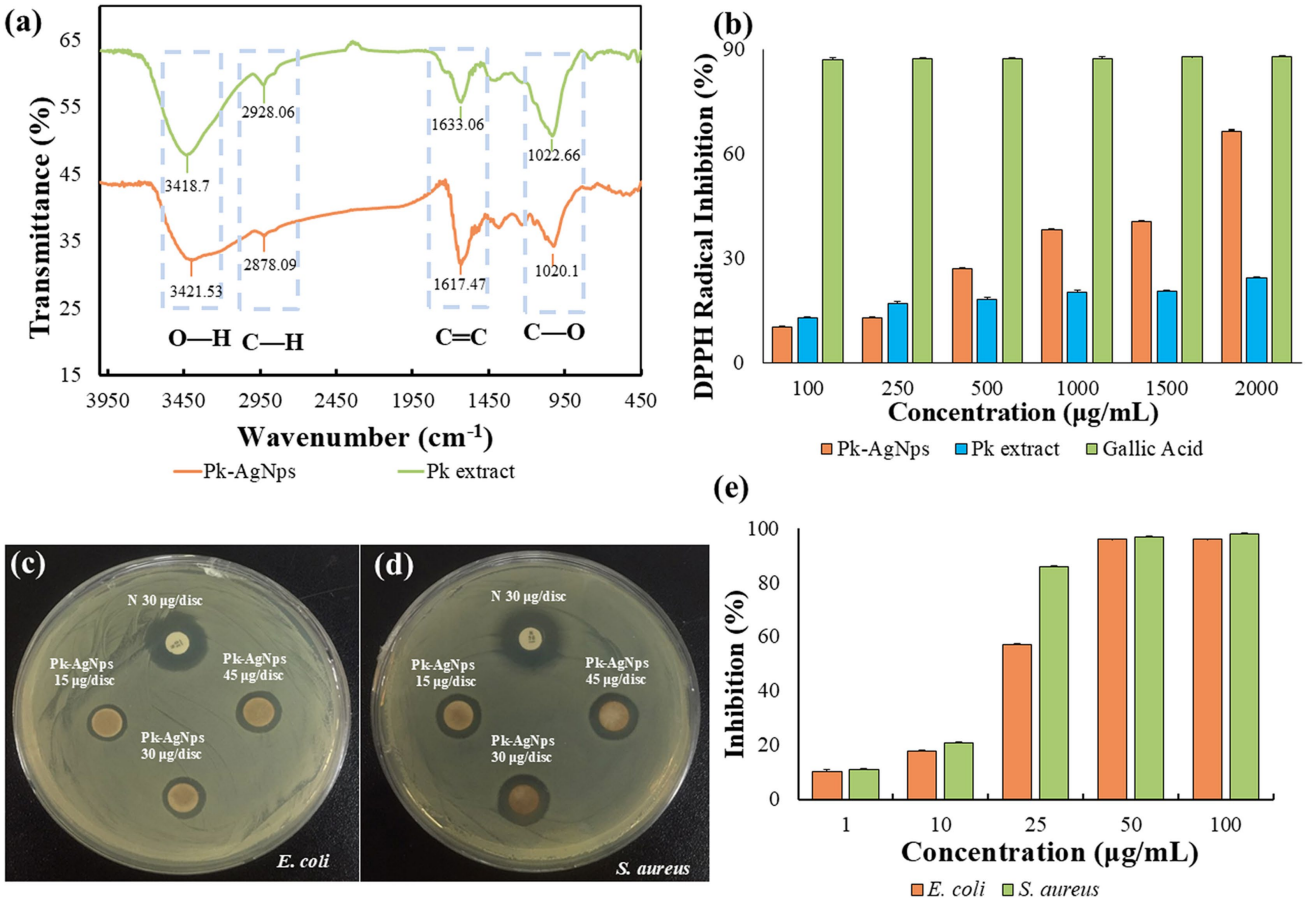
FTIR spectra of Pk-AgNps and plant extract **(a)**, DPPH radical scavenging activity of Pk-AgNps and Pk extract **(b)**. Antimicrobial activity of Pk-AgNps against *E. coli* [ATCC 10798] **(c)** and *S. aureus* [ATCC 6538] **(d)**, presented as inhibition zones determined by the standard disk diffusion method, with neomycin (N30) as a positive control. The MIC of Pk-AgNps against both bacterial strans was quantitatively determined **(e)**. Data represent mean ± SD from three independent experiments.

FTIR spectroscopy was employed to identify the potential biomolecules in the *P. koreana* root extract responsible for the reduction and stabilization of the synthesized Pk-AgNps. A comparative analysis of the spectra of the pure extract and the Pk-AgNps was conducted. As shown in Figure 3a, the broad bands observed in the range of 3,364–3,345 cm^−1^ in the extract spectrum, which correspond to O–H stretching vibrations, were also present in the spectrum of Pk-AgNps. This indicates the involvement of hydroxyl groups from compounds like polyphenols or flavonoids in the synthesis process (33). Furthermore, the spectral regions of 1,626–1,634 cm^−1^ and 1,016–1,067 cm^−1^ in the extract, attributed to C=C stretching vibrations of aromatic rings and C–O stretching vibrations, respectively, were identified (56). These functional groups are characteristic of various phytochemicals, including flavonoids and saponins, which are abundant in *P. koreana* (57).

Crucially, a shift in the position or intensity of these characteristic peaks was observed in the spectrum of Pk-AgNps compared to the pure extract. This shift strongly suggests the coordination of these functional groups (e.g., –OH, C=C, C–O) with the surface of the silver nanoparticles (58). The data imply that the biomolecules containing these groups not only acted as reducing agents for silver ions but also adsorbed onto the surface of the newly formed Pk-AgNps, thereby forming a capping layer that prevents aggregation and ensures colloidal stability. This finding is consistent with the stability observed in the DLS analysis and corroborates the role of the plant extract as an effective stabilizing agent in the green synthesis.

#### 3.2.5 Assessment of antioxidant ability

The antioxidant potential of the biosynthesized Pk-AgNps was quantitatively evaluated using the DPPH free radical scavenging assay. This assay measures the ability of a substance to donate an electron or hydrogen atom to stabilize the DPPH radical, which is reflected by a decrease in absorbance (59). The DPPH scavenging activity of both Pk-AgNps and Pk extract were assessed over a concentration range of 100–2,000 μg/mL, with gallic acid employed as a standard reference antioxidant.

As illustrated in Figure 3b, the Pk-AgNps demonstrated a concentration-dependent increase in DPPH radical scavenging activity. The scavenging rate was lowest at the concentration of 100 μg/mL and progressively increased to its maximum at 2,000 μg/mL. The calculated half-maximal inhibitory concentration (IC50) value for the DPPH scavenging activity of Pk-AgNps was 1,676.77 ± 12.53 μg/mL. In contract, Pk extract exhibited no significant antioxidant activity across the tested concentration range, failing to reach an IC50 value even at the highest concentration of 2,000 μg/mL. This marked difference suggests that the biosynthesis process enhances the antioxidant properties of the plant extract through the formation of silver nanoparticles. Although the absolute scavenging activity of the Pk-AgNps was lower than that of gallic acid at equivalent concentrations, the clear dose–response relationship confirms the intrinsic antioxidant capability of the nanoparticles. This property is particularly relevant for potential applications in functional food systems, where oxidative rancidity is a major concern, as it suggests that Pk-AgNps could be explored as a nanomaterial to enhance oxidative stability (60).

### 3.3 Antimicrobial ability

The antimicrobial activity of biosynthesized Pk-AgNps was evaluated against Gram-negative *E. coli* and Gram-positive *S. aureus* using the standard disk diffusion method, with the broad-spectrum antibiotic neomycin (30 μg/disk) serving as the positive control to validate the experimental system (Figures 3c,d). As summarized in Table 2, Pk-AgNps exhibited a dose-dependent inhibitory effect against both strains within the tested concentration range (15–45 μg/disk). In comparison, neomycin (30 μg/disk) produced inhibition zones of 16 ± 0.33 mm for *E. coli* and 16 ± 0.00 mm for *S. aureus*, confirming the validity of the assay system. Notably, the inhibitory effect was more pronounced against *E. coli*, which showed a zone of 14.0 ± 0.0 mm at 45 μg/disk, compared to 11.7 ± 0.3 mm for *S. aureus* at the same concentration.

**Table 2:** Diameter of inhibition zone (mm) of 30 μL purified Pk-AgNps.

Pathogenic microorganisms	Zone of inhibition (mm)^*^			
	N30 30 μg/disk	Pk-AgNps 15 μg/disk	Pk-AgNps 30 μg/disk	Pk-AgNps 45 μg/disk
*E. coli* [ATCC 10798]	16 ± 0.33	11.33 ± 0.66	12.66 ± 1.33	14.00 ± 0.00
*S. aureus* [ATCC 6538]	16 ± 0.00	10.66 ± 0.88	11.00 ± 0.58	11.66 ± 0.3

* All data expressed as mean ± SD were either calculated from three independent experiments.

MIC values were further determined (Figure 3e), with MIC_90_ values of 25 μg/mL for *E. coli* and 50 μg/mL for *S. aureus*. These results are consistent with previously reported antimicrobial activity of silver nanoparticles (61). The antimicrobial mechanisms of AgNps are multifaceted, released Ag^+^ ions can disrupt respiratory electron transport, inhibit enzymatic activity, and alter membrane permeability (62). Additionally, the nanoparticles may bind to sulfur-containing membrane proteins and intracellular phosphorus compounds such as DNA, and can induce the formation of low-molecular-weight regions within cells, potentially leading to cellular aggregation (63). Overall, AgNp exert their effects through combined mechanisms including membrane damage, ROS generation, and interference with cellular processes (64).

The observed dose-dependent enlargement of inhibition zones confirms the concentration-responsive activity. The stronger efficacy against *E. coli* is likely attributable to structural differences in bacterial cell walls, the thick, multi-layer peptidoglycan of *S. aureus* may hinder nanoparticle penetration, whereas the LPS-rich outer membrane of *E. coli* is more susceptible to disruption by Pk-AgNps, resulting in increased membrane permeability, leakage of cellular content, and ultimately cell death (35).

In summary, Pk-AgNps demonstrate significant and selective antibacterial properties, with particularly potent activity against Gram-negative bacteria, supporting their potential as natural antimicrobial agents in fields such as food preservation.

### 3.4 *In vitro* biological studies of Pk-AgNps

#### 3.4.1 *In vitro* cytotoxicity of Pk-AgNps

The anticancer potential of the biosynthesized Pk-AgNps was investigated *in vitro* against three human cancer cell lines: A549 (lung carcinoma), MCF7 (breast adenocarcinoma), and AGS (gastric adenocarcinoma). The cells were treated with different concentrations of Pk-AgNps for 24 h, and the resulting cell viability was assessed.

**Figure 4:**
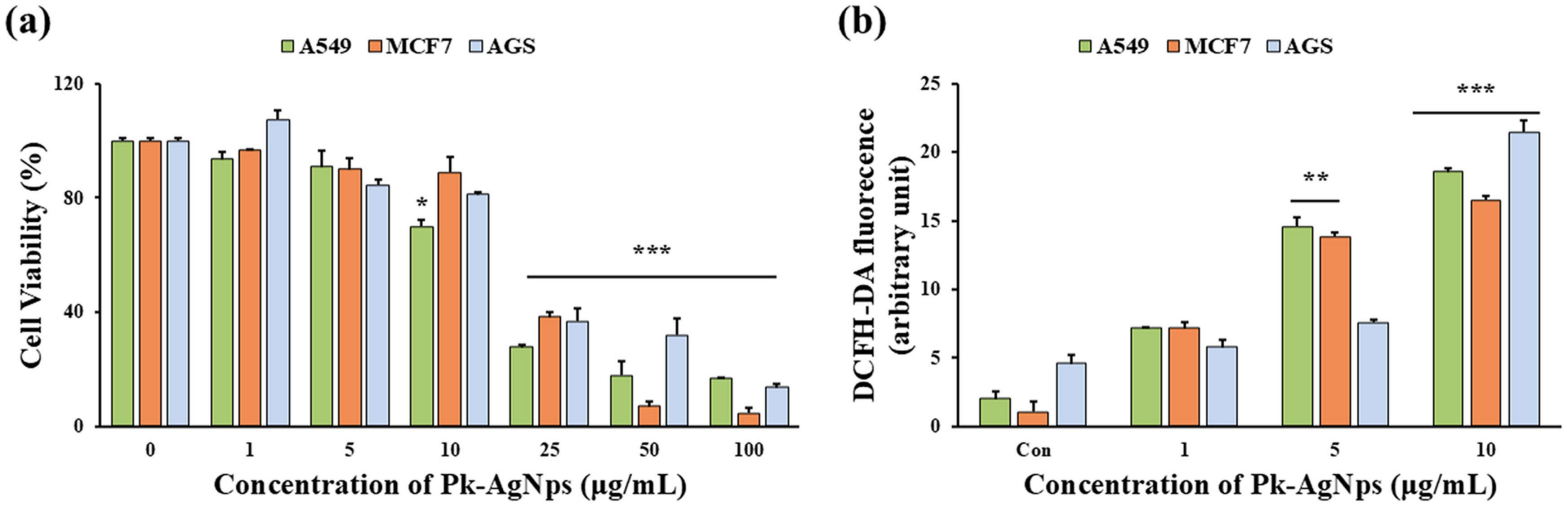
Cytotoxicity of Pk-AgNps in A549, MCF7, and AGS cells for 24 h treatment **(a)**. Intracellular ROS levels in the three cancer cell lines **(b)**. Data represent mean ± SD from three independent experiments. ^*^p < 0.05, ^**^p < 0.01, and ^***^p < 0.001 vs. control.

As summarized in Figure 4a, Pk-AgNps induced a concentration-dependent reduction in cell viability across all three cancer cell lines. The IC50, a key metric for quantifying the potency of a substance where a lower value indicates greater potency, was determined for each cell line. The calculated IC50 values further clarified the differential sensitivity: A549 cells were the most sensitive with an IC50 of 16.75 ± 0.66 μg/mL, followed by AGS at 19.26 ± 0.49 μg/mL, and MCF7 at 21.04 ± 0.27 μg/mL. Consistent with this, the A549 lung cancer cells demonstrated the highest susceptibility, with a decrease in cell viability observed at a concentration as low as 10 μg/mL. A significant cytotoxic effect in all three cell lines was observed at 25 μg/mL.

Furthermore, at nearly all tested concentrations, the viability of A549 cells was the lowest among the three lines, indicating that the A549 cell line was the most inhibited by Pk-AgNps. In the absence of a positive control, the observed cytotoxic effects cannot be directly benchmarked against a standard agent; future studies incorporating such controls would allow for more definitive comparative assessment of potency. The cell viability of A549 was consequently the lowest, underscoring the potent and selective cytotoxicity of Pk-AgNps against this particular lung cancer model.

#### 3.4.2 Intracellular ROS evaluation in different cancer cell lines

To investigate a potential mechanism underlying the observed cytotoxicity of Pk-AgNps, the generation of intracellular ROS was measured in the A549, MCF7, and AGS cancer cell lines following treatment with the nanoparticles. The results demonstrated that exposure to Pk-AgNps induced a concentration-dependent increase in intracellular ROS levels across all three cell lines (Figure 4b). However, the threshold for significant ROS generation varied among the different cell types (65). In line with the cytotoxicity data, A549 and MCF7 cells appeared to be more sensitive to ROS induction by Pk-AgNps. A significant elevation in ROS levels was detected in both A549 and MCF7 cells at a relatively low concentration of 5 μg/mL. In contrast, a significant increase in ROS in AGS cells was only observed at a higher concentration of 10 μg/mL.

The differential ROS response correlates well with the cell viability results, where A549 cells were the most sensitive, followed by MCF7 and then AGS cells. This suggests that the oxidative stress induced by Pk-AgNps is a key contributor to their cytotoxic effects. The accumulation of intracellular ROS beyond a cell’s antioxidant capacity can cause severe damage to lipids, proteins, and DNA, ultimately triggering apoptosis or other forms of cell death (66).

#### 3.4.3 Pk-AgNps induced nuclear damage to cancer cells

**Figure 5:**
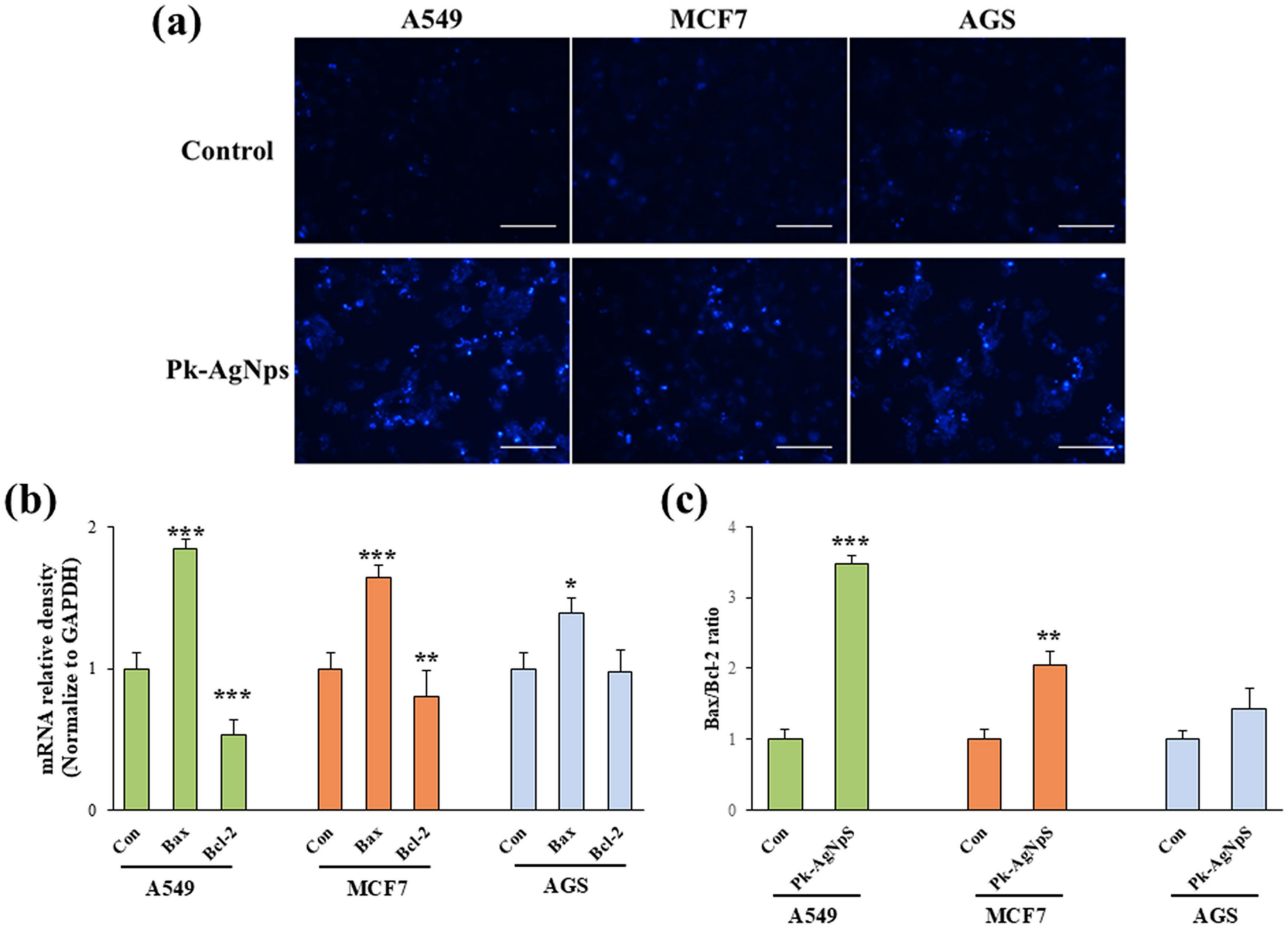
Hoechst staining showing nuclear morphology in A549, MCF7, and AGS cells after Pk-AgNps treatment **(a)**. Scale bar = 10 μm. Gene expression analysis by qRT-PCR: *Bax* and *Bcl-2* expression **(b)**, and *Bax*/*Bcl-2* ratio **(c)** in three cancer cells. Data represent mean ± SD from three independent experiments. ^*^p < 0.05, ^**^p < 0.01, ^***^p < 0.001 vs. control.

To further elucidate the mechanism of cell death induced by Pk-AgNps, nuclear morphological changes were assessed using Hoechst-33342 staining after treating A549, MCF7, and AGS cells with the nanoparticles for 24 h. As depicted in Figure 5a, untreated control cells exhibited normal, uniformly stained nuclei with regular oval or round shapes. In contrast, cells treated with Pk-AgNps displayed classic hallmarks of apoptosis, including nuclear condensation, karyorrhexis, and the formation of apoptotic bodies. These morphological alterations confirm the induction of apoptotic cell death (67).

The extent of nuclear damage was consistent with the cytotoxicity and ROS generation profiles. The A549 cell line, which demonstrated the highest sensitivity in viability assays and the most pronounced ROS generation at low concentrations, also showed the most severe and widespread nuclear damage following Pk-AgNps treatment. A significant number of A549 cells exhibited condensed and fragmented nuclei even at lower concentrations. While MCF7 and AGS cells also showed clear apoptotic features, the effects were generally less severe compared to A549 at equivalent concentrations.

The induction of nuclear damage is a downstream consequence of severe cellular stress, often triggered by elevated intracellular ROS (68). The observed ROS-mediated oxidative stress likely causes DNA damage and disrupts nuclear membrane integrity, initiating the apoptotic cascade (69). The correlation between robust ROS generation and extensive nuclear fragmentation in A549 cells provides a compelling mechanistic link, suggesting that the superior cytotoxicity of Pk-AgNps against this cell line is executed through an efficient ROS-driven apoptotic pathway.

These results demonstrate that Pk-AgNps induce apoptosis in cancer cells, with the most profound effects observed in A549 lung cancer cells.

#### 3.4.4 Cellular response to Pk-AgNps treatment*in vitro*

To elucidate the molecular mechanism underlying Pk-AgNps-induced apoptosis, the expression levels of key genes regulating apoptosis and cell proliferation were analyzed by qRT-PCR. As shown in Figure 5b, qRT-PCR results demonstrated that Pk-AgNps treatment significantly altered the expression of key apoptotic regulators, showing marked upregulation of the pro-apoptotic gene *Bax* alongside pronounced downregulation of the anti-apoptotic gene *Bcl-2* across all three cancer cell lines. This shift in the *Bax*/*Bcl-2* expression ratio represents a pivotal event in mitochondrial-mediated apoptosis (70, 71), with the consistently elevated in *Bax*/*Bcl-2* ratio (Figure 5c) providing molecular substantiation for the observed nuclear fragmentation and apoptotic morphology.

**Figure 6:**
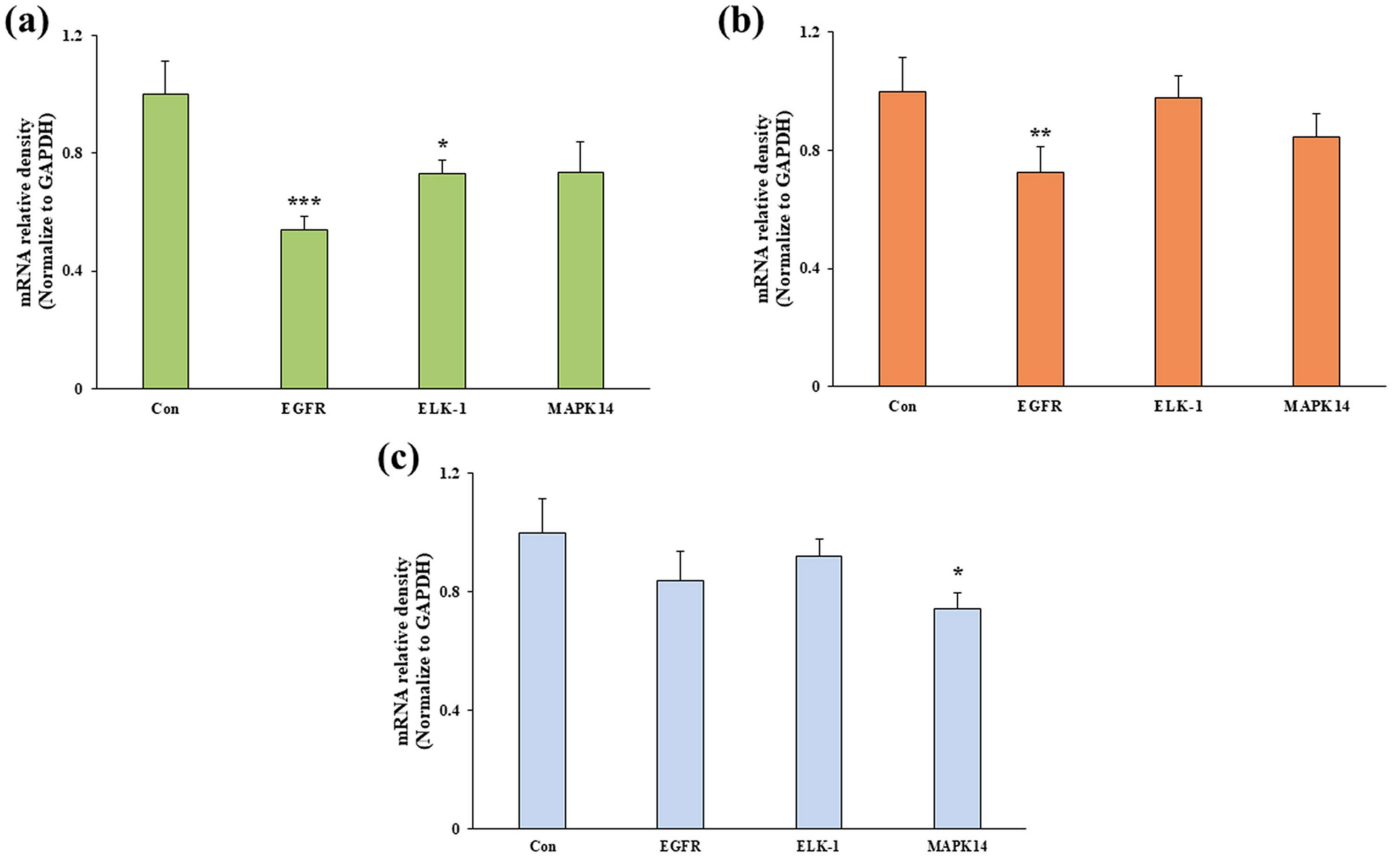
Expression level of *EGFR*, *ELK-1*, and *MAPK14* genes in A549 **(a)**, MCF7 **(b)**, and AGS **(c)** cells following Pk-AgNPs treatment. Gene expression was normalized to *GAPDH* and presented as mean ± SD from three independent experiments. ^*^p < 0.05, ^**^p < 0.01, ^***^p < 0.001 vs. control.

Further investigation revealed that Pk-AgNps effectively disrupted pro-survival signaling pathways through substantial suppression of the EGFR-mediated MAPK cascade. Pk-AgNps treatment significantly downregulated *EGFR* expression in A549, and MCF7 cells (Figure 6), accompanied by coordinated suppression of downstream effectors *ELK-1* and *MAPK14* (*p38α*). The concurrent downregulation of these critical signaling molecules indicates effective disruption of pro-survival pathways (72). The concurrent downregulation of thee critical signaling molecules indicates comprehensive disruption of cellular survival networks (73, 74), while the altered *Bax*/*Bcl-2* ratio promotes initiation of the intrinsic apoptotic cascade (75, 76). This dual mechanism, simultaneously activating mitochondrial apoptosis and inhibiting survival signaling, explains the potent cytotoxicity observed in cancer cells, particularly the enhanced sensitivity of A549 cells. The coordinated transcriptional changes demonstrate the multi-targeted action of Pk-AgNps, highlighting their potential as multifaceted nutritional supplements capable of concurrently modulating multiple regulatory pathways to induce programmed cell death.

Based on the comprehensive findings, this study elucidates the dual mechanism through which Pk-AgNps induce selective cytotoxicity—simultaneously triggering mitochondrial apoptosis and suppressing the EGFR-mediated MAPK survival pathway. These molecular insights provide a mechanistic foundation for the bioactivity observed. Importantly, while the green synthesis of nanoparticles using plant extracts is an established approach, this work specifically utilized the aqueous root extract of *P. koreana* not merely as a reductant and stabilizer, but to generate nanoparticles whose bioactivity may be uniquely enhanced by the synergistic phytoconstituents of this medicinal plant. Beyond successful synthesis, our research provided rigorous multi-technique characterization to define the physicochemical properties of Pk-AgNps. Furthermore, while many studies report isolated biological effects, this work integratively evaluated a triad of relevant bioactivities—antioxidant, antibacterial, and effects on cancer cell models—within a single nanoparticle system. Crucially, the analysis deliberately frames the observed *in vitro* cytotoxic effects within a mechanistic and exploratory context, while highlighting the robust, demonstrated antioxidant and antibacterial properties as the primary basis for proposing practical applications, such as in food preservation. This shift in emphasis from broad biomedical potential to a focused, application-driven evaluation, grounded in a clear molecular understanding, represents a key contribution of this investigation.

## 4 Conclusion

This study reports the green synthesis of Pk-AgNps using an aqueous extract of *P. koreana* roots. Comprehensive characterization confirmed the formation of high purity, spherical, and crystalline nanoparticle. The Pk-AgNps demonstrated significant bioactivity, exhibiting potent, concentration-dependent antioxidant capacity and strong broad-spectrum antibacterial activity against both Gram-positive and Gram-negative bacteria. In preliminary assessments, an inhibitory effect on the viability of cultured cancer cells, particularly A549 cell line, was noted upon treatment with Pk-AgNps, accompanied by observable increases in ROS levels and indicators of mitochondrial apoptosis. These multifunctional properties suggest the potential of Pk-AgNps as a promising candidate for applications in food preservation and as a functional food ingredient.

## Data Availability

The original contributions presented in the study are included in the article/supplementary material, further inquiries can be directed to the corresponding author.
